# Historical Techniques of Lie Detection

**DOI:** 10.5964/ejop.v11i3.919

**Published:** 2015-08-20

**Authors:** Martina Vicianova

**Affiliations:** a Pavol Jozef Šafárik University in Košice, Košice, Slovakia; Aalborg University, Aalborg, Denmark

**Keywords:** lie, lie detection, medieval procedure, phrenology, brain-based lie detection

## Abstract

Since time immemorial, lying has been a part of everyday life. For this reason, it has become a subject of interest in several disciplines, including psychology. The purpose of this article is to provide a general overview of the literature and thinking to date about the evolution of lie detection techniques. The first part explores ancient methods recorded circa 1000 B.C. (e.g., God’s judgment in Europe). The second part describes technical methods based on sciences such as phrenology, polygraph and graphology. This is followed by an outline of more modern-day approaches such as FACS (Facial Action Coding System), functional MRI, and Brain Fingerprinting. Finally, after the familiarization with the historical development of techniques for lie detection, we discuss the scope for new initiatives not only in the area of designing new methods, but also for the research into lie detection itself, such as its motives and regulatory issues related to deception.

Social psychology defines deception as: “a communicator’s deliberate attempt to foster a belief or understanding in others which the recipient considers to be untrue” (DePaulo et al., 2003). Similarly, dictionaries use these very features to define lying and deception. For example, according to the Oxford Dictionary of English (Stevenson & Soanes, 2010), deception is “a statement that deviates from or perverts the truth”. Deception is a pervasive, and some would argue a necessary, phenomenon in human communication, yet its very action stirs up moral indignation and rage. As a result, for as long as there have been lies, there have been methods of lie detection. The concept of lying and the ability to do so have reached a new level with technological advances that have moved lie detection from the realm of fire and water to EEGs, FACS and functional MRIs. But the question remains as to whether human beings are better equipped to detect lying than centuries ago. Are the public, legal and scientific communities’ obsessions with finding liars actually yielding better results, or are they merely sugarcoating the same techniques with fancy machinery? This article will attempt to provide a general overview of the literature and thinking to date about the concept of historical and contemporary detection of lying. This will provide an introduction for a historical and critical review of lie detection techniques primarily in, but not limited to, Western culture.

The purpose of this article is to contribute to scientific reflections on lie detection through analytic methods used until now and thus emphasize the need to advance the creation of new methods for the detection of fraud in which the psychological, spiritual and social variables (based on psychophysiological measures) are taken into consideration. Moreover, the article aims to educate and stimulate the reader about the complex nature of lying, while raising questions about whether technology has really advanced the art of detecting deception and whether some of the techniques mentioned will ever consistently meet legal standards for scientific evidence.

The reader should note that this subject is so vast that some topics are mentioned only briefly or even excluded.

## Lie Detection

Lie detection is a part of numerous criminal, medical or legal professions. Police officers are challenged by deception especially in the determination of facts in crimes that have been committed. Judges and lawyers seek justice in legal disputes and medical specialist demand the truth for accurate diagnosis and appropriate treatment of patients. The following part is an overview of the most commonly used methods of lie detection.

### Early Methods of Lie Detection

Ford (2006) reported that one of the first methods to prove the veracity of a statement uttered by the accused was described in China circa 1000 BC. The person suspected of lying was required to fill his/her mouth with a handful of dry rice. After a while, s/he was to spit out the rice. If the expectorated rice remained dry, the suspect was found guilty of fraud. This method was based on the physiological principle and the assumption that experiencing fear and anxiety is accompanied by decreased salivation and a dry mouth. The works of contemporary authors (Matsumoto, 2009; Praško, 2011) imply that fear paralyzes us and is physically reflected in an increased heart rate and a mental sense of hopelessness. The somatic expression of anxiety and fear include changes in behaviour associated with the feeling of a dry mouth. The symptomatology is similar to manifestations of depression, panic disorder and the like (Höschl, Libiger, & Švestka, 2004). Given the fact that the aforementioned knowledge about the physiological manifestations of anxiety were not known at that time and thus not taken into account, the majority of prisoners, regardless of whether they had actually committed a crime or lied, were executed. Several centuries later Erasistratus, Greek physicist and physician (300-250 B.C.), tried to detect deception by measuring the pulse. This same technique re-emerged as a part of testing with polygraph in 1921 (Trovillo, 1939).

#### The trial by ordeal method

The historical writings of various European countries more often mention a technique known as *trial by ordeal* - or the *Judgments of God* (Apfel 2001; Holák, 1974; Sullivan, 2001). This was another method used by authorities in the interest of detecting lies and finding the truth. It was used to prove the truth of a claim of an accused person by a specific act that the person had to go through. Based on its favourable or unfavourable outcome, the claim was accepted as true or false.

The rationale (and hence the court’s argument) was based on the belief that God would not let a righteous man suffer and injustice prevail. For example, in the territory of present day Slovakia, the first courts were established in the 11th century. They concerned either a one-sided substantiation of the truth by the accused person, or a double-sided one, when one was subjected to the judgment of God (Holák, 1974). The one-sided judgment of God was conducted by a water test or a fire test. The water test was carried out by ​​using either hot or cold water. When using the hot water test, the accused was ordered to place the hand into a cauldron with boiling water and hold it there for a specified time. If the hand in boiling water showed no traces of scalding or small blisters, it represented a sign of the accused person’s claim to be true. A variation of this test involved the accused retrieving a ring or stone out of the cauldron of boiling water.

The test based on cold water included throwing the accused person into the water in a roped sack. If the tested person emerged at the surface in a short time, it signified that “not even water accepts him/her” – or more precisely – servants of the devil (hence liars too) rejected baptism and that is why water cannot accept him/her (Sullivan, 2001, p. 213).

Regarding the hot water method, there were individuals who doubted the credibility of this procedure. In 1593, in the Netherlands, the court turned to a university and asked for examination of the water test to determine its appropriateness as a lie detection technique. In this case, rationality won and the test was disapproved (Apfel, 2008). However, application of the test with cold water remained popular until the 18th century as evidenced by court records in Vojtka on the Danube, when 70 women were subjected to this test (Holák, 1974).

In the case of using fire as a proving method, the accused was compelled to carry a hot piece of metal for a certain distance or walk across burning embers. The accused was considered innocent if either no wounds appeared or they healed quickly.

Sometimes the court turned to the use of consecrated meal. The examining person was a priest who, at the end of worship, gave the accused a piece of dry bread and a piece of hard sheep’s cheese. The accused was exhonorated if he or she managed to swallow in one bite without difficulty. However, if the person choked or suffocated, the test brought a guilty verdict. There are some similarities between this method and the Chinese use of rice. In China, as well as in Europe, people attempted to identify lies through methods using the mouth. However, the Chinese method’s verification was based on objective knowledge of physiological manifestations of fear – in case of fear, the mouth stays dry.

Methods based on God’s judgment ceased to exist past the 15th century, and only the cold water test mentioned above remained. It was used mainly for the substantiation of witchcraft. People gradually realized that the guilt or innocence of a person could not be detected using various “experiments” based on magic or divine forces possessing the power to protect the innocent. The change in the assessment of truth and deception came gradually through the development of various scientific fields.

#### Phrenology and graphology

In 1870, Franz Joseph Gall discovered a new possibility of detecting deception through recognition of emotions of the accused. The theory was elaborated and further improved in cooperation with his pupil Spurzheim. The point of their interest was examination of specific areas of the brain assuming the existence of relations between different abilities and skull shape (Rafter, 2005). The main ideas of their theory pointed to the brain as the central organ of the mind which can perceive individual emotions such as emulousness, ambition, destructiveness and, among the many others, the tendency to lie, and to engage in criminal behavior. The more active parts of the brain are well recognizable from the contour of the skull (these areas were more convex or concave). It was assumed that the relative size of each area can be enlarged or reduced by training and self-discipline. Gall became a pioneer in mapping the human skull and this newly-created scientific discipline was named phrenology. Gall often made public appearances demonstrating various criminals with shaved heads and emphasized the “anomaly” on the skulls. Through phrenology, he tried to determine liars randomly chosen from the audience. His services were also occasionally used in legal disputes to determine which party was lying.

In the field of criminology, phrenology helped to spread the belief that delinquent behavior (together with lying) should be the subject of scientific study. It strengthened the medical model of criminal behavior according to which the behavior of some perpetrators may be affected by brain malfunctions. By virtue of this idea, many crimes were reassessed saving a multitude of mentally ill people from being unfairly sentenced (Trovillo, 1939). Although phrenology fell into oblivion and was discredited, Gall’s work rendered an important service by reminding researchers that the human body is affected by environmental factors and together they build an entity of mutual relations (Hall & Lindzey, 2002).

Simultaneously with phrenology, graphology began to spread and in 1875 started to be considered a useful scientific method of lie detection (Schönfeld, 2007). Its origins are associated with an effort to detect forged signatures which led to the scientific analysis as we know it nowadays. Its founder, J. H. Michon, assumed that some peculiarities of handwriting may relate to certain personality traits. Following the analysis of handwriting, graphology attempts to identify a personal writing movement through which the nature of the writer may be manifested (Schönfeld, 2007). The excitement around graphology as a method of lie detection ended after the First World War. During the war graphology was deemed an appropriate means of verifying the authenticity of documents and signatures. However, graphology was not acknowledged as an appropriate tool for lie detection. Nowadays, this method is used in various areas such as employment profiling (to do a personality profile) or psychological analysis (used alongside other projective personality assessment tools), (Poizner, 2012; Thomas, 2001).

### Contemporary Methods of Lie Detection

#### The polygraph

After phrenology, in 1881, the first modern lie detection device called Lombrosso’s Glove was created by an Italian criminologist, physician and anthropologist Cesare Lombrosso. He attempted to measure changes in the accused person’s blood pressure which were recorded on a graph or chart. This sophisticated technology was improved on during the First World War by William M. Marston and was elaborated into its final version only after the war in 1921. This device served to record changes in blood pressure and changes in breathing while giving testimony (Trovillo, 1939). A few years later, John Larson and Leonard Keele designed a psychiatric device called “Cardio-Pneumo Psychograph” also known as a polygraph or a lie detector (Lewis & Cuppari, 2009). This polygraph recorded respiratory rate, blood pressure changes, and changes in galvanic skin response (bioelectric reactivity of the skin). Given the fact that studies (Lewis & Cuppari, 2009) point out to the ratio between thoracic and diaphragmatic breathing as a sensitive indicator of stress and emotional change (male and female breathing in these indicators usually differ), modern polygraphs measure respiratory rate of chest and abdomen separately which leads to a significant increase in the diagnostic value of measurement. The basis for evaluating the outcome of a polygraph is in the relationship between physiological changes which manifest when a person is not telling the truth. These changes can be observed and measured by the polygraph using skin conductance, blood pressure, heart rate and respiration. Unfortunately, bodily changes can vary and are also produced by states other than lying (Brewer & Williams, 2005). In this kind of testing, two types of questions were used – the Control Questions Test (CQT) and the Guilty Knowledge Test (GKT). The standard polygraph is often the CQT since it is most often used in criminal investigations. The CQT often asks the suspect two types of questions – control questions and relevant questions. The control questions are related to the suspect’s crime investigation, but not specifically to the crime. This test measures the suspect’s detailed knowledge of a crime that he or she does not want to share. For example, the polygraph examiner might discuss with a suspect several different types of cars, one of which was actually used in committing the crime (Lewis & Cuppari, 2009).

In the late 1990’s, the polygraph began to be used in the United States not only by the police but also for verifying the reliability of the public safety employees and managers (verifying the veracity of the information provided about themselves in the CV, previous employment, etc.). Due to its growing popularity and recurring inaccurate results, the test of reliability was performed on polygraph. National Academies of Science (NAS) indicated the reliability of polygraph as 81% - 91% (National Research Council, Committee to Review the Scientific Evidence on the Polygraph, 2003, p. 4). These results were supported by researchers such as Fiedler, Schmid, and Stahl (2002), Bartol and Bartol (2004), Grubin and Madsen (2005), Grubin (2008), Lewis and Cuppari (2009), Ginton (2013). For example, the signs of nervousness, fear and emotional disturbances occur not only in people who reported false information, but also in people who tell the truth. In conclusion, it can be stated that the polygraph does not detect lies but instead measures physiological responses postulated to be associated with deception. None of these responses are specific to deception, nor are they necessarily always present when deception occurs. However, when used by well-trained examiners and in conjunction with other techniques, it appears to offer a useful adjunct in identifying those who attempt to deceive (National Research Council, Committee to Review the Scientific Evidence on the Polygraph, 2003 p. 7).

Observation of nonverbal expressions and Voice Stress Analysis. The desire to detect lies is not reflected only in the use of various technical equipment. Observation and attention focused on some specific behavioral expressions have also played important roles. Darwin (2002/1872) described Duchenne’s work from 1862 which supposes the possibility of revealing the truth by observation of facial expressions. A smile which is the result of experiencing happiness is manifested by constriction of zygomatic major muscle (musculus zygomaticus major) causing the corners of the mouth to lift. In case of electrical stimulation of this muscle, the smile appears to be unnatural. Similarly, this applies to the circular muscles in the eye (orbicularis oculi) which, when constricted, pull the face slightly higher and depress the eyebrows. These two muscles can reveal the true emotional state since their activity can be purposely controlled only with great difficulties, remarked Charles Darwin in 1872 (Ford, 2006).

By the mid-1960s, Ekman initiated a series of cross-cultural studies focusing on face expressions, emotions and gestures. In addition to his basic research on emotions and their expression, he had also been studying deceit. In 1991, Ekman conducted a study in which he focused on the ability to identify lies by individuals of various professions. It concerned mainly professions in which one encounters lies more frequently. The participants were members of secret services, psychiatrists, judges, policemen, personnel operating polygraphs, and a group of university students. The individuals were asked to describe the changes in behavior, in facial expressions, and in the voice intonation of a woman giving testimony, and, based on these clues, come to a conclusion as to whether her statement was true or false. The most successful group proved to be the group of secret service agents. The authors saw a relationship of this outcome in the fact that most of these agents had an experience with the protection of important statesmen where, during public displays, they had to rely on nonverbal expressions of people in the crowd. A negative correlation with age was discovered, in the sense that participants younger than 40 years scored better. The researchers assumed that junior colleagues have more actual experience in the field, whereas senior colleagues are more likely to be engaged in administrative work. Kohnken’s experiment (1987; as cited in Vybíral, 2003) with respect to age of the investigators reported the opposite results. In the course of the training of police officers, a positive age correlation with success to detect false eyewitnesses was found. Senior investigators were more successful in detection. Further results of Ekman’s study give evidence of the poor success of judges and psychiatrists in the detection of lies. The judges’ failure was explained by pointing out that they do not see the face of a person giving a testimony because the witness often sits in a position where the judge cannot see the face. Judges tend to focus their attention on listening and writing notes. Psychiatrists did not consider it important to recognize the initial lies supposing that the lie would eventually come to the surface (Ekman & O'Sullivan, 1991). Interpreting the results, it should be taken into consideration that the above-mentioned research work was predominantly focused on the ability of a trustworthy delivery of emotions (videotaped nurses) and the related specific type of lies – concealing emotions – which can greatly affect the possibility to generalize the outcomes. Ekman highlighted the difference between what people think and what they know. This difference is associated with the fact that people often overestimate their ability to detect lies (McNeill, 1998).

Craig, Hyde, and Patric (1991) conducted research to help clinicians identify distortions of the pain experience, such as willfully minimizing pain displays (masking) or exaggeration of such behaviors (simulating). Authors identified the facial movements associated with masking and simulating, as well as genuine pain. In response to this study, Galin and Thorn (1993) conducted research which focused on the identification of false expressions of pain using the method called FACS (Facial Action Coding System). FACS is a research tool useful for measuring any facial expression a human being can make. FACS is an anatomically based system for detailed description of all observable facial movements. This method was invented by Ekman in 1979. Later, this method was improved and renamed as Ekman Micro Expression Training Tool. Manual for this method has been designed to be self-instructional. That is, people would read the manual, practice with video images, and eventually take a final test for certification (Ekman, 2015).

In addition, Ekman (1996) presented six interpretations as to why we are not successful in lie detection. One of the reasons is the evolutionary lack of facilities for authentic disguise as well as for the detection of lies. People in communities constantly lived close together and did not have many opportunities to cover cheating and, as mentioned at the beginning of the article, the discovery of lies lead to the application of extreme sanctions. Living conditions have significantly changed and society provides more possibilities for lying, at times it almost encourages its members to lie or to use half-truths (e.g., advertising sector, trade and business). Yet, we still possess little sensitivity to detecting lies (Vybíral, 2003). An individual, according to Ekman (1996), is generally predetermined to trust which also makes life simpler. As for the fourth factor, Ekman (1996) mentions the desire to be deceived or not knowing the truth. It includes situations in which we do not want to know anything about some facts, thus we do not ask questions (e.g., in relationships). Another of Ekman’s explanations is based on the conclusions of Goffman (1974, as cited in Ekman, 1996) who states that it is important for us to be socially accepted – to be affable – rather than tell the truth (so-called courtesy lies). The last explanation directly concerns professionals who deal with detecting lies. As we have seen in the study of Ekman (1996), none of these results showed the ability to accurately recognize lies. In this case, an appropriate method has proven to be the training by FACS (Facial Action Coding System) allowing successful lie detection by examining emotional expressions in 70% of cases (Matsumoto, Hwang, Skinner, & Frank, 2011).

The manifestation of experienced emotions is related to another technique called Voice Stress Analysis (VSA). Voice stress analysis (VSA) is accomplished by measuring fluctuations in the physiological micro tremor present in speech. A micro tremor is a low amplitude oscillation of the reflex mechanism controlling the length and tension of a stretched muscle caused by the finite transmission delay between neurons to and from the target muscle. Microtremors are present in every muscle in the body including the vocal chords and have a frequency of around 8–12Hz. During times of increased stress, this microtremor shifts in frequency. This change in frequency transfers from the muscles in the vocal tract to the voice produced. On the basis of these findings, the VSA is regarded as a suitable means, for example, the detection of false statements. In a comparative study, Patil, Nayak, and Saxena (2013) state that micro tremor frequency of Voice Stress Analysis (VSA) technology can identify emotional stress better than the polygraph. Patil et al. (2013) plans further reliability test of the VSA detecting false testimonies in the area of justice.

#### Brain-based lie detection

Since the 1980’s and the onset of neuroscience, completely different views on the possibility of detecting lies at the highest level of mental processes have emerged through the medium of measuring brain activities such as transcranial magnetic stimulation – TMS, functional magnetic resonance imaging – fMRI, positron emission tomography – PET and Brain Fingerprinting (EEG wave). At the time of this writing, papers on this topic have been published by Bles and Haynes (2008), Ganis, Kosslyn, Stose, Thompson, and Yurgelun-Todd (2003), Langleben et al. (2002), Lee et al. (2002), and Spence et al. (2001). In our article, we describe the most frequently used methods: Brain Fingerprinting, PET, EEG and fMRI. Guevin (2002) described the first Brain Fingerprinting method invented by Donchin and his student Farwell in 1990. Brain Fingerprinting is a way of detecting a specific EEG (electroencephalograph) wave. The theory is that the brain processes known and relevant information differently from the way it processes unknown or irrelevant information (Farwell & Donchin, 1991). The brain’s processing of known information, such as the details of a crime stored in the brain, is revealed by a specific pattern in the EEG (Farwell, 1994; Farwell & Smith, 2001). Farwell's brain fingerprinting originally used the well-known P300 brain response to detect the brain’s recognition of the known information (Farwell, 1995; Farwell & Donchin, 1986, 1991). Later, Farwell discovered the P300-MERMER (“Memory and Encoding Related Multifaceted Electroencephalographic Response”), which includes the P300 and additional features and is reported to provide a higher level of accuracy and statistical confidence than the P300 alone (Farwell, 1994; Farwell, 1995; Farwell, 2012; Farwell & Smith, 2001). In peer-reviewed publications, Farwell and colleagues report less than 1% error rate in laboratory research (Farwell & Donchin, 1991; Farwell & Richardson, 2006) and real-life field applications (Farwell & Smith, 2001; Farwell, 2012). In an independent research, Iacono (1997, as cited in Allen & Iacono, 1997) confirmed Farwell´s results (Allen & Iacono, 1997). Despite of the results, the method of brain fingerprinting exhibits some disadvantages. For these techniques to be of use in the detection of criminal offenses, researchers must have a sufficient amount of information about the event and the perpetrator. This is necessary in order to be able to document the suspect´s EEG patterns when the correct answer is provided. It is also conceivable, especially with the intensity of media coverage, that a suspect may possess information about a crime without being the perpetrator. Brain Fingerprinting is much more expensive and requires more time and preparation than a standard polygraph; this, along with Farwell´s patent, has limited the extent to which this procedure can be used.

Other possibilities of using the graphical brain imaging for the purpose of lie detection are functional magnetic resonance imaging (fMRI – functional magnetic resonance imaging) and positron emission tomography (PET), which focus on the activity of the central nervous system (brain and spinal cord) and not the peripheral nervous system (neurons). In another study, Langleben et al. (2002) used BOLD (blood oxygenation level-dependent) fMRI in an attempt to localize changes in regional neuronal activity during deception. They studied 18 students and subjected them to a version of the GKT involving playing cards. They found a cluster, extending from the right anterior cingulate gyrus to the medial aspect of the right superior frontal gyrus that was significantly different between the two conditions of telling the truth and lying. This cluster has been reported to be associated with response conflict and open-ended responses, as well as some executive functioning tasks, such as decision-making and task performance (Carter et al., 1998). The anterior cingulate cortex, more specifically, is involved in emotional processing and conflict resolution (Tancredi, 2004).

A group of researchers at Harvard (Ganis et al., 2003) used BOLD fMRI to compare the brain activation in three scenarios: truth, spontaneous lies and memorized lies. They found that both types of lies elicited more activation in the anterior prefrontal cortices bilaterally, involved in retrieving memory (Fletcher & Henson, 2001). They also found that spontaneous lies preferentially activated the anterior cingulate cortex as compared with memorized lies. This is consistent with Langleben’s et al. (2002) findings in the study, and may be related to the conflict associated with inhibiting the truth.

Experiments continued whereas fMRI focused on observation of the temporal lobes where a part of the cerebral cortex (FFA - fusiform face area) becomes activated when a person looks at a human face. Further in the front is an area (PPA - parahippocampal place area) which activates once a person observes buildings and objects. During a random projecting of faces and different objects, it is possible to detect, with an 80% success rate, what was actually spotted (Kahnwisher, 2009, as cited in Koukolík, 2011). With this assumption, experiments were launched inducing situations in which respondents were asked to lie. Investigation of the brain brought seven areas that were activated more than others when telling a lie. 90% of cases were identified correctly.

Koukolík (2011) draws attention to the fact that it is not an experiment that answers the question lie - truth, but rather the question of which areas of the brain are active when a person is lying. It is most important that the experimenters knew who was really lying and who was not since the participants were asked to lie. The study of Monteleone et al. (2009) analyzed the data of involved participants and the results showed that none of the parts of the cerebral cortex can be used for accurate lie recognition in individual cases. One of the alternatives seemed to be monitoring of the medial prefrontal cortex. This was successful in 71% of the participants. Kozel, Johnson, Mu, Grenesko, Laken, & George (2005) obtained similar results. In his study, he attempted to explore the so-called simulated crime in which the participants were asked to steal an object. During the MRI examination, he was asked to lie about this event. The misleading reply stimulated mainly the prefrontal cortex. In another study, a group was asked to shoot from a gun and consequently lie about this act. In this case, only the anterior part of the cerebral cortex and the left lateral visual area were activated. The source of these differences might be in the different nature of the lie. The alteration of the task meant processing of diverse stimuli, whether acoustic or visual. Responses to the activity also differed; it incorporated either a verbal or a practical response – pressing the button, choosing the right card, playing the scenario. Every given task is processed in a different way.

All fMRI studies on lie detection typically describe young and healthy adults. However, BOLD activity is known to be altered with age (Buckner, Snyder, Sanders, Raichle, & Morris, 2000; D’Esposito, Deouell, & Gazzaley, 2003), in patients with cardiovascular diseases (Pineiro, Pendlebury, Johansen-Berg, & Matthews 2002; Röther et al., 2002), and those suffering abuse (Levin et al., 1998; Sell et al., 1997). Other published studies examining brain function during deception have demonstrated that the results of these methods are not sufficiently precise and lack strong empirical foundation (Greely & Illes, 2007; Porter, ten Brinke, & Gustaw, 2010; Spence, 2008; Wolpe et al., 2005 as cited in Vrij, Mann, & Leal, 2013). Specifically, Spence (2008) points to problems with replication, large individual brain differences, and unspecified brain regions associated with truth telling. Also, brain activity when lying depends on the situation.

Another limitation of techniques in the field of lie detection is the human brain itself. It processes all that passes through the perceptual field not only from the outside, but also from inside. It is irrelevant to what extent this activity will penetrate into consciousness. The accusation, whether rightly or wrongly, brings the brain to the limit situation: energizes memory, attention, emotional level and decision-making ability. All of these changes can be recorded using functional magnetic resonance or through a polygraph. However, it is possible to “hide” what is going on in one’s brain during the task solution by occupying the brain with completely different activities (e.g., mathematical operations, memories), a technique called self-defense (Koukolík, 2011). This is the first range of issues which discredits brain imaging methods. The second problem arises from experiments that are purely laboratory and the participants are largely a specific sample of students (Department of Psychology or Medicine). Their results may not correspond with the results of other tested groups. As for the third problem, it arises from the fact that the experimenters are asked to deceive so this is not considered a spontaneous lie. The possibility to avoid this would be not to follow instructions. The experimenter should intervene only if the participant asks for it. The fourth issue relates to the diversity of situations and with different types of deception. Different people with the same kind of fraud can have a different type of attitude. Therefore, the personality and attitude of an individual need to be taken into account.

## Conclusions

In this paper we attempted to create an overview of techniques applied for lie detection from the available resources. Our aim was to compose a chronological description of techniques for lie detection that have been most frequently used primarily in Western culture. A relatively large part of this article has been devoted to unscientific methods from the Middle Ages called *trial of ordeal* or the *judgments of God* that were part of the judicial system for centuries and uncomplex, in some cases subjectively judging the lies (guilt – innocence) of prisoners. We intended to bring the reader closer to this site of the human desire for knowledge of the truth which often overlapped with the desire for power and control over others.

In reference to the fact that the most develop methods to detect deception began to emerge in the 80s of last century, the main part of the article primarily focused on their description. We presented methods that are in the process of scientific examination such as Functional magnetic resonance imaging – fMRI, positron emission tomography – PET and Brain Fingerprinting (EEG wave), but also those which have already become available to the general public and are used, for example, in the business sphere for job interviews, such as Voice Stress Analysis and Facial Action Coding System. In addition to these methods, new ways are beginning to appear and various procedures so as to reveal liars; for example, “Ways to catch the liar” (within the internet search engine, more than 10 million references are available about procedures for an effective lie detection). Interest in this topic has an upward trend and the growing number of sources complicates the orientation in this area. Our intention was to compose a concise and informative framework describing common methods used in lie detection and thus facilitate the reader’s general orientation in this field. Furthermore, we point out the need to develop new alternative methods for the detection of deception that would be less dependent on external factors and would be based rather on psychophysiological measures (e.g., the expectation that lying may cause guilt and fear) reflected as a response in the autonomic nervous system.
